# Co-Solvent Selection for Supercritical Fluid Extraction (SFE) of Phenolic Compounds from *Labisia pumila*

**DOI:** 10.3390/molecules25245859

**Published:** 2020-12-11

**Authors:** Shazana Azfar Radzali, Masturah Markom, Noorashikin Md Saleh

**Affiliations:** 1 Department of Chemical and Process Engineering, Faculty of Engineering and Built Environment, Universiti Kebangsaan Malaysia (UKM), Bangi 43600, Selangor, Malaysia; pjkkp@ukm.edu.my (S.A.R.); cespro@ukm.edu.my (N.M.S.); 2 Research Centre for Sustainable Process Technology (CESPRO), Faculty of Engineering & Built Environment, Universiti Kebangsaan Malaysia (UKM), Bangi 43600, Selangor, Malaysia

**Keywords:** green technology, SFE, co-solvent, antioxidant, phenolic acid

## Abstract

A preliminary study was conducted to study the effects of different types and concentrations of co-solvents based on yield, composition and antioxidants capacity of extract prior to optimization studies of supercritical fluid extraction (SFE) of *Labisia pumila* (locally referred to as ‘kacip fatimah’). The following co-solvents were studied prior to the optimization of supercritical carbon dioxide (SC–CO_2_) technique: ethanol, water, methanol, as well as aqueous solutions of ethanol–water and methanol–water (50% and 70% *v*/*v*). By using the selected co-solvents, identification of phenolic acids (gallic acid, methyl gallate and caffeic acid) was determined by using High-Performance Liquid Chromatography (HPLC). Then, the antioxidant capacity was evaluated by using three different assays: total phenolic content (TPC), ferric reducing/antioxidant power (FRAP) and free radical-scavenging capacity of 2,2-diphenyl-1-picrylhydrazyl (DPPH). SC–CO_2_ with 70% ethanol–water co-solvent was superior in terms of a higher combination of phenolic compounds extracted and antioxidants capacity. Overall, SC–CO_2_ with co-solvent 70% ethanol–water technique was efficient in extracting phenolic compounds from *L. pumila*, and thus the usage of this solvent system should be considered for further optimization studies.

## 1. Introduction

**Figure 1:**
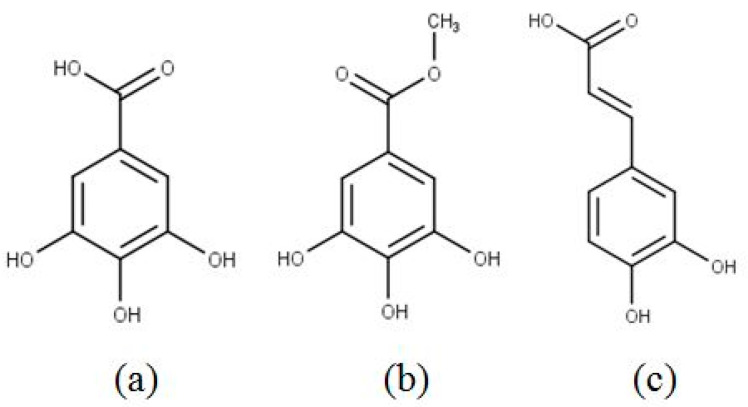
Chemical structure of phenolic compounds (**a**) gallic acid, (**b**) methyl gallate, (**c**) caffeic acid.

*Labisia pumila* (Blume) Fern.-Vill. synonym *Marantodes pumilum* (Blume) Kuntze or commonly known as ‘kacip fatimah’ is recognized in the Malaysian National Key Economic Areas (NKEA) to be developed as a herb for commercial purposes. It is one of the most popular herbs found in Southeast Asia and receives high demand from the locals in Malaysia, Thailand and Indonesia. The popularity of ‘kacip fatimah’ is demonstrated by the emergence of several commercial products, including tablets, capsules, tonics and health drinks that contain this herb. Recent studies on *L. pumila* demonstrated that it contained potent phenolic compounds that have been proven to have many biological effects, such as strong antioxidant, anticancer, anti-viral, anti-bacterial and anti-inflammatory properties [1,2,3]. Arguably, this could at least be partly due to the presence of important phytochemicals, such as gallic acid (3, 4, 5-trihydroxybenzoic acid) and other phenolic compounds. Most phytochemicals present in the extract of *L. pumila* are phenolic components, including phenolic acids, flavonoids and other detected compounds (ascorbic acid and carotenoids) [4,5]. Chemical structures of active phenolic compounds, namely gallic acid, methyl gallate and caffeic acid are presented in Figure 1 [5,6].

Previously, many conventional methods (decoction, maceration, and soxhlet) were intensively conducted by other researchers to extract the plant phenolic compounds [1,3,4,7,8]. Maceration was extensively applied in medicinal plant research as it is the easiest technique and a better option for some herbal constituents that are sensitive to heat. Other studies have also focused on modern methods for phenolics extraction, such as microwave-assisted extraction (MAE), pressurized hot water extraction (PHWE), pressurized liquid extraction (PLE) and ultrasound-assisted extraction (UAE) [3,7,9,10]. Nevertheless, the amount of phenolic compounds in those studies was comparatively low as compared to the traditional techniques, which are economically unprofitable and the selectivity of specific compounds extracted still remained uncertain. Moreover, limited study concerning the phenolic compounds and antioxidant capacity of this plant extract is available. One recent study [8] reported the effect of solvents (water, ethyl acetate, hexane and ethanol) on the extraction of gallic acid from *L. pumila* leaves by using solid–liquid extraction (SLE). Nevertheless, no studies were conducted on other solvents or analysis of other components besides gallic acid [8] and to the study knowledge, the application of SFE has not been reported for this herb. Some researchers postulated that SFE offers recoveries that are effective or even better than those of conventional chemical extraction techniques [11]. The extraction efficiency of SC–CO_2_ could be increased by modifying the solvent selectivity, while the solubility of polar phenolics in the mixture could be improved by introducing co-solvents of different types and concentrations. In addition, the comparatively low solubility of polyphenols in SC–CO_2_ requires high pressures (>40 MPa) or the addition of polar modifiers to enhance its dissolution in the mixture with simultaneous improvement in the extraction yields [12,13,14,15].

Additionally, most herbal products that contain *L. pumila* extract do not have information about the active ingredients present in the product. The identification and quantification of phenolic substances with high biological effects present in the herb is essential for verification and standardization purposes of *L. pumila*-based products. Prior to identification and quantification, extraction of active constituents from *L. pumila* is a crucial stage before it can be developed further as functional foods or supplements. In herbal processing, the understanding of solvent effects is very significant for the screening, solvent selection and separations steps (extraction, fractionation, purification, and chromatography). By understanding the property of solvents, components (solute) and solvent–solute interaction, an effective and fast fractionation and isolation of targeted compounds can be achieved [16]. Therefore, this study evaluated the effects of different types and concentrations of co-solvents on the amount of important phenolic compounds content (gallic acid, methyl gallate and caffeic acid) in the *L. pumila* leaves, including the antioxidants capacity of the extracts by using SC–CO_2_ owing to the abundance of this herb in Malaysia. Then, the best co-solvent will be used for further optimization of the SFE process.

## 2. Results and Discussion

### 2.1. Effects on Extraction Yield

From Table 1 and Table 2**,** results indicated a wide range of extraction yield for different solvents (2.79–14.71%). The 50% (*v/v*) ethanol–water solvent in both extraction techniques, exhibited the highest total yield (Table 1 and Table 2). The same trend was also observed by a recent study, whereby 50% (*v*/*v*) ethanol–water solvent through the cold maceration method gave the highest extraction yield of 7.6% 14.3% and 17.4% for *L. pumila, Phyllanthus niruri* and *Orthosiphon stamineus*, respectively [17]. In cold maceration, 50% ethanol–water solvent yielded the highest percentage (6.95% *g*/*g*), followed by 70% ethanol–water, 50% methanol–water, 70% methanol–water, methanol, water, ethanol and the least extract was obtained when *n*-hexane was used (1.18% *g*/*g*). The results showed that there was no significant difference (*p* > 0.05) between the yield obtained from methanolic and ethanolic solvents. Water extract also gave a comparable yield of 3.05 ± 0.08%, which indicated that polar compounds were easier to be extracted as compared to nonpolar compounds. Ethanol, methanol and water have hydroxyl groups which can form hydrogen bondings with the solute. However, the higher polarity and shorter chain of water cause it to be more effective in extracting the solute [18]. Therefore, by adding water to the organic solvent, extraction yield for both methanolic and ethanolic solvents could be improved. For the SC–CO_2_ technique, 50% ethanol–water solvent also gave the highest yield and the least extract yield was recovered when co-solvent was not used (2.95% *g*/*g*).

Researchers also noticed that different colors of the extract were observed, whereby aqueous solutions of ethanol–water and methanol–water (50% and 70% *v*/*v*) extract colors were dark brownish (in both methods), brownish with a tinge of green in pure ethanol and methanol extract, and brownish in water. The presence of chlorophylls in the extract is a possible explanation for the tinge of green extract color. The color of extract from nonpolar solvents (*n*-hexane) and (pure carbon dioxide) was observed to be pale yellowish. In the herbal extraction, *n*-hexane was normally used to remove nonpolar unwanted glycosides and lipids [20,21].

### 2.2. Effects on Extract Composition

The composition of components content extracted is presented in Table 1 and Table 2. The polarity index of all co-solvents and co-solvent mixtures used in this study is also shown in Table 1 and Table 2. Water had the highest polarity index (9.0), but did not yield the highest combination of phenolic compounds due to low solubility [22,23]. In contrast, ethanol and methanol had an almost similar polarity index (5.2–6.6) and high yield. Nevertheless, aqueous solutions of ethanol–water and methanol–water (50% and 70% *v*/*v*) were solvents of choice for yielding higher content of phenolic compounds. Even though in both extraction techniques the 50% ethanol–water solvent exhibited the highest total yield, the targeted phenolic compounds (gallic acid, methyl gallate and caffeic acid) showed a greater tendency to dissolve in 70% ethanol–water as compared to other solvent mixtures studied. The large amounts of phenolic content in the 70% ethanol–water extracted solution were due to the optimal combination of organic solvent and water.

For the cold maceration method, the highest gallic acid content (0.17% *g*/*g* extract) was obtained when water was chosen as a solvent. (Table 1). Findings were also in good agreement with those of Yeop et al. [24], which reported that the ultrasonic *L. pumila* extract in water yielded about 29% higher gallic acid content than that of 10% in ethanol–water solvent. Paini et al. also noticed that the higher vapor pressure and viscosity of ethanol, with respect to water, could reduce the implosion force of the cavitation bubbles [25]. Therefore, the implosion caused a less efficient solid material disruption, which was important for the release of gallic acid from the matrix [26]. In regard to polarity, water has higher polarity than ethanol. Under these circumstances, gallic acid which is classified as a polar phenolic compound, that is more soluble in water or solvent mixture of higher polarity than ethanol. Whereas, the highest methyl gallate content (0.04% *g*/*g* extract) were obtained when 50% ethanol–water was used as the solvent. In the maceration extraction method, the best solvent for extracting caffeic acid was 70% (*v*/*v*) ethanol–water solvent, which gave the highest caffeic acid content (0.81% *g*/*g* extract) (Table 1). 

In the SC–CO_2_ technique, the extraction process by using 70% ethanol–water as co-solvent exhibited the best gallic acid, methyl gallate and caffeic acid contents (Table 2). Generally, the findings showed that for both techniques, the mixture of 70% (*v*/*v*) ethanol–water solvent gave the optimum extraction yield and component content. Normally, water is added to organic solvents for two reasons; firstly to enhance the solubility of phenols by weakening the hydrogen bonds in aqueous solutions, and secondly to improve the permeability of leaves tissue, and thus allows better mass transport via molecular diffusion [27,28]. However, water has much greater surface tension and viscosity, which are undesired in the extraction process as solvent absorption into the active sites would be hindered in the matrix [29]. Nevertheless, the cohesive energy and dielectric constant are significantly greater, and thus, water molecules are more strongly bonded to the very polar phenolic compounds. For SFE, which uses water as a co-solvent (Table 2), the low extraction yield might be due to the leftover water that remained in the extractor vessel, as the highly water-soluble analytes would be partitioned in the aqueous phase. The remaining water would disturb the analyte flow into the fluid mixture [28]. A larger amount of water in the solvent might also influence the identification and quantification of phenolics and interfere with sugars, organic acids and soluble proteins [30,31,32].

To our study knowledge, existing literature showed that the range of gallic acid content in three varieties of *L. pumila* Benth (*Pumila, Alata, and Lanceolata*) leaf from Malaysia were between 0.022% *g*/*g* to 0.185% *g*/*g* [5,7]. Previously, Md Salehan et al. [8] reported that the maximum gallic acid content in *L. pumila* was observed at 50 °C after 6 h of UAE with 0.185% *g*/*g* gallic acid. Interestingly in this study, by using the SC–CO_2_ technique with 70% ethanol–water as co-solvent, the gallic acid content was detected to be higher than reported (0.30% *g*/*g*) (Table 2). Meanwhile, the range of caffeic acid content from methanolic extract of stem, root and leaf of three *L. pumila* Benth varieties (*Alata, Pumila* and *Lanceolata*) were found between 0.002% *g*/*g* to 0.012% *g*/*g* [5]. The caffeic acid amount extracted in this work was also higher (1.11% *g*/*g*) than previously recorded. Nevertheless, to the best of the study’s knowledge, there is no information available on the range of methyl gallate content in *Labisia pumila* leaves. The variations in the bioactive constituent yields (individual phenolic contents) of the extracts reported might attribute to different extraction techniques and polarities of different components that existed. Other than that, a slight difference in the morphological location of solute in matrix, harvest season, collected biomass geographical area as well as ecological and climate system could also modify the extract constitution [33], and thus sometimes result in a wide range of polyphenols content.

**Figure 2:**
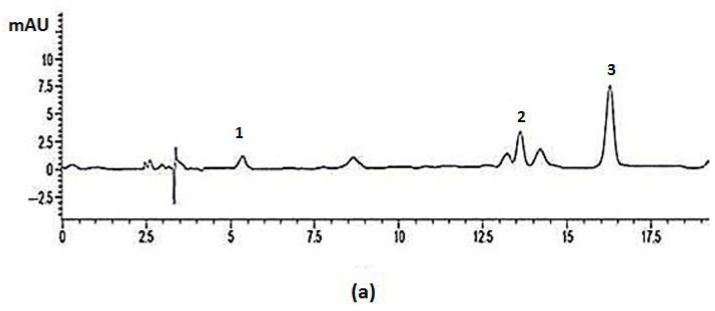

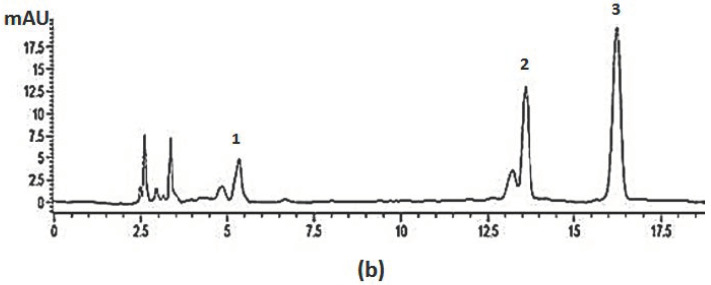
HPLC analysis of phenolics compounds extracted (**a**); maceration technique in 70% (*v*/*v*) ethanol–water from leaves of *Labisia pumila*, (**b**) SC–CO_2_ with 70% (*v*/*v*) ethanol–water as a co-solvent, at 60 °C and 20 MPa from leaves of *Labisia pumila*, (1) Gallic acid, (2) Methyl gallate, (3) Caffeic acid.

Another interesting aspect of this study was the comparison between the phenolics compounds profiles obtained from the maceration technique in 70% ethanol–water and SC–CO_2_ extracts by using the same solvent as a modifier. As can be seen in Figure 2 the difference in profiles might be caused by variations in polarity and solubility of individual constituent content in the extracts. The solubility parameters of both substances must be similar for a component to dissolve in the solvent. The solute solubility in a solvent depends on the hydrogen and electrostatic bonding, polarity (dipole moment) and conformation (dihedral angle) [16]. The tendency of solvents to accept/donate H^+^ (electrophilic) and to accept/donate electron (nucleophilic) with the compounds is also important. All these factors are essential to explain the solvent–solvent and solvent–solute interactions. From the findings, we can also conclude that components containing carboxylic acid and hydroxyl groups are preferably extracted by water through hydrogen bonding. The ester and carboxylic acid groups have the nucleophilic ability (electron donor) in the carbonyl group and can interact with both alcohol and water [16]. Nonetheless, since the phenolic compounds comprise more than one functional group (Figure 1), each component was selectively soluble in different ethanol to water ratios. For example, water is preferred for the extraction of gallic acid but the addition of ethanol 50%, (*v*/*v*) is a more preferable option for the extraction of methyl gallate.

Nevertheless, the presence of three main component peaks was consistently detected in both HPLC chromatograms and other *L. pumila* extracts. Gallic acid, its derivative (methyl gallate) and caffeic acid had a retention time of 5.4 min, 13.6 min and 16.2 min, respectively (Figure 2). The polarity of caffeic acid was slightly below those of gallic acid and methyl gallate. The free phenolics (gallic acid) were relatively polar, while more complex compounds like methyl ester (methyl gallate) were relatively less polar as compared to free phenolics. Solute solubility in the solvent mixtures could be roughly predicted by the presence of functional groups in the chemical structure of phenolic compounds [16]. Caffeic acid and gallic acid contain carboxylic acid groups and are most soluble in polar solvents like water. Methyl gallate does not have this functional group, and thus the polarity was dependent on the next polar group (hydroxyl) and (ester) (Figure 1). Nevertheless, the proportion and number of these groups as compared to the more polar functional groups might play crucial roles in decreasing or increasing the solubility in a solvent [29]. Therefore, gallic acid is possibly the most polar, followed by methyl gallate and finally caffeic acid.

### 2.3. Effects on Phenolic Content Analysis and Antioxidant Activity

The total phenolic content (TPC) in the extracts of *L. pumila* is shown in Table 3. The total antioxidant activity was assessed based on DPPH free radical inhibition test and the ferric reducing/antioxidant power (FRAP) test. The total antioxidant activity (DPPH and FRAP results) of this plant also showed the same trend with the TPC and individual phenolics content results, whereby the lowest IC_50_ and highest FRAP results were obtained from 70% ethanol SC–CO_2_ extracts (Table 3). Most individual phenolics detected from this plant (Table 1 and Table 2) also showed the same trend with the TPC result, whereby the highest components content was obtained from 70% ethanol extracts. The FRAP value and percentage of DPPH inhibition (IC_50_) showed the same trend, in which a positive correlation existed between the reducing capability of the extracts and antioxidant activity. Yildrim et al. [34], found that the antioxidant power was related to phenolic antioxidant activity. Meanwhile, *n*-hexane and SC–CO_2_ extracts yielded nearly the same and the lowest antioxidant power (the highest IC_50_ value and lowest FRAP value) due to the low solubility of the nonpolar solvents (Table 3). In contrast, 50% (*v*/*v*) ethanol in water extract had moderate phenolic amounts and antioxidant capacity. The existence of the relations demonstrated that the composition of *L. pumila* extracts reported earlier may account for their antioxidant capacity.

The results also indicated that all plant extracts had a noticeable effect on DPPH scavenging activity (IC_50_ ≤ 50.0 µg/mL) as compared to the values obtained for other reference standards, except for *n*-hexane and carbon dioxide extracts (Table 3). The value of IC_50_ was the concentration of antioxidant (µg/mL) that can inhibit 50% free radicals. The value of IC_50_ was obtained from the intersection of the line between 50% barrier power with concentration axis, then substituted value of *y* = 50 to Equation (1); *y* = a In(*x*) ± c. The value of x denotes the value of IC_50_. According to standard IC_50_ value, sample with IC_50_ >150 µg/mL had weak antioxidant, sample with 101 µg/mL < IC_50_ < 150 µg/mL, it had medium antioxidant. Whereas, sample with 50 µg/mL < IC_50_ < 100 µg/mL had strong antioxidant. Meanwhile sample with IC_50_ < 50 µg/mL had very strong antioxidant [35,36].

(1) Y = a ln(x)±c,

where *Y* is the 50% of the maximum percentage inhibition, and *x* is the concentration at which 50% inhibition of the oxidation can be achieved. The values of a and c are generated by the excel operation.

According to a study conducted by Huang et al. [37], a high yield of phenolic contents did not necessarily come with high antioxidant power as the antioxidant activity might also be dependent on other factors like the phenolic component structure, hydroxyl, carboxylic acid, flavone and their interactions in emulsions play an important role in the antioxidant capacity [37]. Of all phenolic acids studied (reference standards), gallic acid was found to be the most active antiradical agent (IC_50_ = 13.32 ± 0.37 µg/mL), followed by methyl gallate (IC_50_ = 16.21 ± 0.46 µg/mL) and caffeic acid (IC_50_ = 19.83 µg/mL), which showed excellent inhibitory activity against DPPH radicals as compared to BHT (IC_50_ = 57.37 ± 0.39 µg/mL) and ascorbic acid (IC_50_ = 41.83 ± 0.28 µg/mL) (Table 3). This was in accordance with the findings of Karamac et al. [38] which found that gallic acid possessed a stronger antioxidant capacity than other phenolic acids. Gallic acid with a carboxyl group and three hydroxyl groups showed a higher level of free-radical sequestering than methyl gallate, with a carboxylic acid ester group and three hydroxyl groups (Figure 1). According to Gorden et al. [39], the methoxy group of methyl gallate may also hinder the scavenging effect of the hydroxyl groups by intramolecular or intermolecular hydrogen bonding. Whereas, caffeic acid with a carboxylic acid group and two hydroxyl groups showed lower scavenging activity than methyl gallate. The result of the present study supported the previous study that phenolic acid compounds, presenting a catechol unit (two hydroxyl groups on the phenolic ring), exhibited lower antioxidant properties than those which presented a pyrogallol unit (three hydroxyl groups on the phenol ring) [40]. Previous research studies indicated that substitution with a hydroxyl group was found to be more effective than the methoxy group [41].

### 2.4. Effects on Plant Matrix

**Figure 3:**
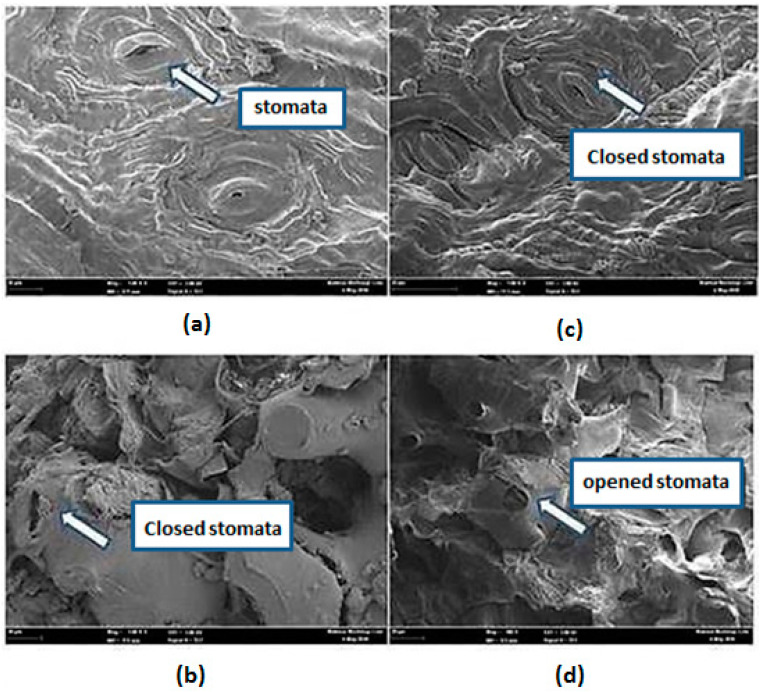
Field-emission Scanning Electron Microscope (FESEM) of the *Labisia pumila* leaves powder surface (**a**); before extraction (with 1000× magnification), (**b**) after maceration in 70% ethanol–water (with 1000× magnification), (**c**) after SC–CO_2_ extraction (pure CO_2_) (with 1000× magnification), (**d**) after SC–CO_2_ extraction using 70% ethanol–water as a co-solvent (with 500× magnification).

Matrices are known to affect extractability in SFE as well as in traditional extraction methods. Matrix swelling was suggested to be a significant factor in modifier-enhanced supercritical fluid extraction [42]. Therefore, an investigation into the influence of the co-solvents/modifiers and the supercritical fluid on the matrix is significant to identify potential phenomena affecting extractions. In this study, the *L. pumila* leaves powder was observed by FESEM to investigate the effect of different extraction techniques and polar co-solvents on the physical structure of fine powder. A similar magnification factor was difficult to achieve since particle size distribution was employed (no fixed particle size), and thus the analysis of the same plant particles before and after extraction was impossible. The stomata were ovoid in shape and covered with epicuticular waxes present on the leaf surface (Figure 3a). Based on Figure 3b, a severe fracture was not detected during the maceration technique in 70% ethanol–water, except a few slight ruptures on the surface of the sample. Figure 3c shows that in SC–CO_2_ extraction (pure CO_2_), the removal of the waxy materials (crystals) outside the leaves was incomplete. 

Nonetheless, by SC–CO_2_ extraction with 70% ethanol–water as a co-solvent, swelling and softening process of membranes, cellular structure and dilation of intercellular channels were observed in Figure 3d. The swelling observed was probably due to the interactions between the polar modifiers and polar plant material. The polar *L. pumila* leaf matrix adsorbed co-solvents of similar polarity and swells. These swelling results gave accessibility to areas of entrapment or adsorption sites. At the same time, the waxes were completely removed and leaves shrunk. The shrunken leaves indicated that the inner materials were extracted by a mixture of SC–CO_2_ with 70% ethanol and water. As expected, the effectiveness of solvating power produced by the supercritical solvent was clearly seen in Figure 3d. This helped in the solubilization processes of the targeted component out of the vegetal matrix. Most plant phenolic acid derivatives are deposited in vacuoles [43]. The targeted compounds were intracellular compounds and they were not freely available. During the extraction process, molecular diffusion occurred once the solvent penetrates the cellular materials. Then, the solvent will dissolve the solute and brings out the desired compounds from the plant matrix.

## 3. Materials and Methods 

### 3.1. Sample Preparation 

The plant material, *L. pumila* was purchased from Batu Pahat, Johor. The plant identification was verified by a botanist (Dr Shamsul Khamis) from the Department of Biological Sciences and Biotechnology, Faculty of Science and Technology, Universiti Kebangsaan Malaysia (UKM). The voucher specimen was deposited at the Herbarium of UKM (voucher specimen number of *L. pumila var. pumila* = UKMB 30007/SM s.n.). Prior to the experiment, the *L. pumila* leaves were cleaned by using tap water and dried at room temperature until the moisture content was constant (6% *w/w*). Then, the leaves were blended and sieved with a particle size of ~0.8 mm and kept at 4 °C in the fridge. The particle size was determined in the range of 0.8mm to 0.5 mm by sieving with a standard sample sieve and a sieve shaker. A scanning electron microscope (SEM), (Hitachi S-3400N, High Technologies America Inc., Chatsworth, CA, USA) was used to measure the particle shapes. Most solvents and chemicals used, such as methanol, ethanol and hexane (Fisher Scientific, Waltham, MA, USA) were of analytical grade. The acetonitrile used was of high-performance liquid chromatography (HPLC) grade (Merck Chemicals GmbH, Darmstadt, Germany).

### 3.2. Solvent Extraction

Pulverized leaves *of L. pumila* were submersed in the solvent for 8 h. The sample-to-solvent ratio of each mixture was set at 1:10 (sample:solvent). Several organic (4) and organic–aqueous solvents (4) of various polarities were used, as presented in Table 1 and Table 2. To maintain the sample-to-solvent ratio, the mixture was stored in a sealed bottle to prevent evaporation. Aluminum foil was also used to wrap the outer surface of the bottle to provide a complete barrier to light throughout the extraction process. Then, the sample was centrifuged with a speed of 5000 rpm for 10 min in a Thermo Scientific, Sorvall Biofuge Primo R (Hamburg, Germany) centrifuge machine to separate a heterogeneous mixture of solid and liquid after the extraction process. Extracts were dried in an FX2–2 model air-circulating oven (Sheldon Manufacturing, Cornelius, NC, USA) at 40 °C and collected until a thick and viscous paste or powder of extract was visible. Later, all extracts were left to cool at room temperature before being gravimetrically weighed to determine the yields. Then, the extracts were kept at 4 °C prior to analysis for the determination of bioactive components.

### 3.3. Supercritical Fluid Extraction (SFE)

**Figure 4:**
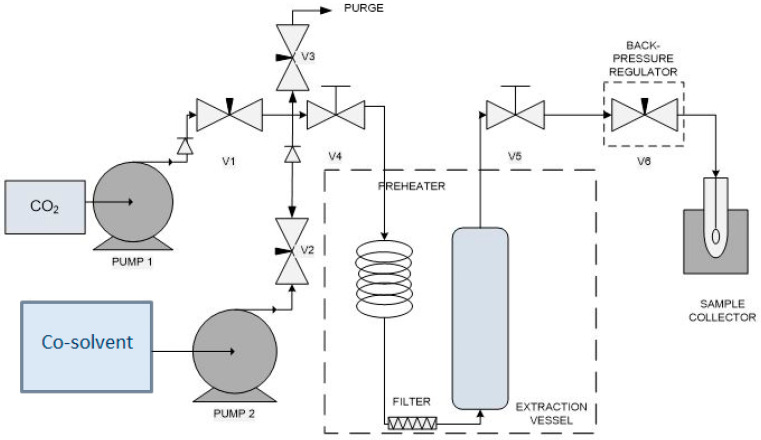
Design of the experimental system used for the supercritical fluid extraction system with co-solvent [29].

Different co-solvents and the effects of their concentration on the extraction yield were investigated by applying SFE with CO_2_ on the sample. Figure 4 illustrates the SFE system which comprised BP-1580-81 model back-pressure regulator (BPR) (JASCO Corporation, Tokyo, Japan), PU-2080 model CO_2_ pump (JASCO Corporation, Hachioji, Japan), Series III solvent pump (Lab Alliance, Syracuse, NY, USA), 682-8 model pressure transmitter (Dwyer Instrument, Michigan, IN, USA), extractor vessel enclosed in an FX2–2 model air-circulating oven (Sheldon Manufacturing, Cornelius, NC, USA) and sample collector. Commercial grade liquefied CO_2_ (99.9%) was purchased from Linde, Malaysia. The CO_2_ was chilled to −2 °C by using a chiller (Protech Electronic, Seri Kembangan, Malaysia) to retain its liquid state before it was pumped into the extractor. The *L. pumila* powder was filled in the extractor which was consisted of a high-pressure stainless steel vessel. The two ends of the extractor were blocked with glass wool to hold the sample and avoid sample entrainment. The SFE process was controlled by needle valves and a back-pressure regulator was used to sustain the system pressure.

Seven types of co-solvents (water, 50% (*v*/*v*) methanol in water, 50% (*v*/*v*) ethanol in water, 70% (*v*/*v*) ethanol in water, 70% (*v*/*v*) methanol in water, pure ethanol and methanol) were studied. The parameters were set at an operating pressure of 20 MPa and a temperature of 60 °C. The effect of co-solvent was studied at a moderate pressure of 20 MPa as at this pressure, the rate of extraction was expected to be moderate. A higher extraction rate might be applicable if the moderate extraction rate was effective. The flow rate of the co-solvent was set to achieve the desired modifier to a CO_2_ concentration of 10% (*v*/*v*). Five grams (±0.05) of the *L. pumila* powder was extracted in the static mode for 30 min (without co-solvent), followed by a dynamic extraction at a solvent flow rate of 4 mL/min for 4 h. The crude extracts were collected every 30 min and then put into an air-circulated oven (Shel Lab, Cor, USA) at 45 °C for about 15–30 h to eliminate the residual co-solvent. All extract fractions were then allowed to cool at room temperature before gravimetrically weighed to calculate the extract yields. Then, the dried extracts were stored in the dark at 4 °C until subsequent analysis.

### 3.4. Determination of Component Analysis

The phenolic acid content in *L. pumila* leaves extracts was determined by using the gallic acid, caffeic acid and methyl gallate standards (Sigma-Aldrich Corporation, Cornelius, MO, USA). All phenolic acid standards were dissolved in 50% ethanol and prepared in a concentration range of 0.1–0.5 mg/mL. Then, all *L. pumila* extract fractions were suspended in 50% ethanol at concentrations of 1–5 mg/mL. For reference, standard solutions were injected at different concentrations in triplicate and linear regression analysis on the data of peak area versus concentration was carried out. Data for the standard curve was obtained with a coefficient of determination, *R*_2_ = 0.994, for gallic acid. The linear regression analysis data of methyl gallate and caffeic acid also demonstrated a good linear relation with *R*_2_ of 0.998 and 0.999, respectively. The satisfactory reproducibility with relative standard deviations (RSDs) was less than 10%. The chromatographic peak identities were determined based on the comparison of their spectra and retention times against the known standards. To determine the solvent retention time, a single injection of solvent (blank) was carried out. The contents of methyl gallate, gallic acid and caffeic acid in the extracts fractions were calculated according to the corresponding peak areas and injected concentrations.

### 3.5. HPLC Analysis

The HPLC analysis was performed according to an established previous method [29] by using the HPLC technique that was equipped with a UV-vis detector (Agilent Technologies, Waldbronn, Germany) and an autosampler with some modifications. A reversed-phase C18 Genesis column with 250 × 4.6 mm i.d. and 4 μm particle diameter (Jones Chromatography, Mid *Glamorgan*, UK) was used for analysis. The separation was achieved by a flow rate of 1mL/min with 0.1% phosphoric acid in water (solvent A) and acetonitrile (solvent B) with a gradient of solvent B: 8–22% (35 min), 22–8% (10 min). The injection volume was 20 μL. The individual polyphenols were detected at the maximum absorption wavelength in the mobile phase: gallic acid (270 nm), methyl gallate (280 nm), and caffeic acid (340 nm). The unknown compounds were also co-chromatographed with known standards. All extract and standard solutions were ultra-sonicated at 40 °C for 30 min to ensure the complete dissolution of all solids and remove air bubbles before analysis by HPLC. Each standard and sample was filtered with a nylon syringe filter (pore size of 0.45 μm). The mobile phase of phosphoric acid and acetonitrile were prepared, degassed and injected through the chromatographic column.

### 3.6. Total Phenolic Content (TPC) Assay

The total phenolic content (TPC) of *L. pumila* extracts was done by using the Folin-Ciocalteu reagent (FC) as described by Singleton and Rossi [44]. A calibration curve was prepared by using a standard solution of gallic acid (20 μg/mL, 40 μg/mL, 60 μg/mL, 80 μg/mL and 100 mg/L, *R*^2^ = 0.970). Properly diluted *L. pumila* extracts solution (20 µL) was mixed with 100 µL of FC reagent in the dark. After the reagent stood for 3–8 min at room temperature, 80 µL of sodium carbonate solution (7.5% *w/v*) was added. The solutions were mixed and allowed to stand in the dark for 2 h at room temperature for the reaction to occur. The absorbance at 765 nm was measured. The results were expressed on a fresh weight basis as mg gallic acid equivalents (GAE)/g sample.

### 3.7. DPPH Free Radical-Scavenging Assay

The antioxidant capacity of *L. pumila* extract was measured by the 1,1-diphenyl-2-picrylhydrazyl (DPPH) assay [45] with slight modifications. The 0.1 mM of DPPH solution was prepared by diluting 1 mg of DPPH in 25 mL of methanol. Each extract was prepared with different concentrations (20 μg/mL, 60 μg/mL, 100 μg/mL, 200 μg/mL, 300 μg/mL, 400 μg/mL and 500 μg/mL). A mixture of 100 μL of DPPH and 50 μL of each extract was transferred into a 96-well microplate (in triplicate). The mixture was shaken for 2 min to mix the solution and kept in the dark (sealed with parafilm) for 30 min incubation before the absorbance at 517 nm was measured against a control solution of methanol and DPPH without extract. Gallic acid, methyl gallate, caffeic acid, butylated hydroxytoluene (BHT) and ascorbic acid were used as the positive controls (reference radical scavengers). The ability of positive controls and extracts to scavenge free radical was calculated by using the following formula:

*I*% = [(*A*_control_* − A*_sample_)/*A*_control_] × 100%. (2)

*A*_control_ is the absorbance of 0.1 mM of DPPH with methanol and *A*_sample_ is the absorbance of the *L. pumila* extracts and positive controls solutions. All results were interpreted by IC_50_ value (Equation (2)). The linear regression analysis data of all standards (gallic acid, methyl gallate, caffeic acid, (BHT) and ascorbic acid) also demonstrated a good linear relationship with *R*^2^ of 0.979, 0.9873, 0.9971, 0.9877 and 0.9794, respectively. The concentration of antioxidant needed to decrease the initial DPPH concentration by 50% (IC_50_) is a parameter widely used to measure the antioxidant activity [46]. A higher IC_50_ value corresponds with lower antioxidant power.

### 3.8. Ferric Reducing/Antioxidant Power (FRAP) Assay

The FRAP assay was done according to an established method developed by Benzie and Strain [47] with some modification. FRAP reagent was freshly prepared by mixing 5 mL 2,4,6-tris(2-pyridyl)-1,3,5-triazine (TPTZ) solution (10 mM) in 40 mM hydrochloric acid solution with 5 mL FeCl_3_·6H_2_O solution (20 mM) and 50 mL acetate buffer solution (0.3 M, pH 3.6) and incubated at 37 °C after mixing. A calibration curve was prepared by using an aqueous solution of ferrous sulfate (FeSO_4_ 7H_2_O at 200 µM, 400 µM, 600 µM, 800 µM and 1000 µM, *R*^2^ = 0.948). Properly diluted *L. pumila* extract (50 µL) was mixed with 1.5 mL of FRAP reagent under dark conditions. The absorbance at 593 nm of 200 µL of the mixture was determined against a blank. FRAP values were expressed on a fresh weight basis as micromoles of ferrous equivalent Fe (II) per gram of sample.

### 3.9. Statistical Analysis

All analyses were performed in triplicate and the results were presented as mean ± *SD*. Statistical analyses were performed by using the statistical package SPSS Version 22.5. Differences at *p* < 0.05 (95% confidence level) were considered to be significant. Analysis of variance (One-way ANOVA) was done to compare different groups of samples, while post-hoc test was used for paired comparisons. An independent *t*-test was used when two groups of samples were compared. In this study, the IC_50_ values were calculated by using Microsoft Office Excel 2010. The IC_50_ value for each sample was determined graphically by plotting the percentage disappearance of DPPH as a function of the sample concentration.

## 4. Conclusions

In light of these experiments, a selective, fast and environmentally friendly SFE technique was proposed for extracting phenolic acids from *L. pumila* leaves. It can be concluded that the types of solvents and their concentrations affect the extraction yield and antioxidant activity. A positive relation between the phenolic content and the antioxidant capacity can be seen from this study: the higher the phenolic content of the plant, the higher the FRAP value and DPPH inhibition (lower the IC_50_ value). The SC–CO_2_ with the use of a modifier is a greater alternative to the traditional chemical solvents for phenolic extraction as it has a higher extraction yield and higher quality of extracts at shorter extraction time. Therefore, the findings in this work may serve as a useful guide to select a suitable co-solvent, which is used to maximize phenolic acid extraction from *L. pumila* and other plants. Based on the above results, 70% ethanol–water was chosen as the best co-solvent and would be used in the optimization study. Further study on the effect of different SC–CO_2_ operating parameters that would be investigated in the optimization studies is important to obtain the final optimum yield and phenolics content with reduced processing steps. From a green technology point of view, the SC–CO_2_ *L. pumila* extracts have potential as a natural source of antioxidants that could be used in different fields (cosmetics, foods, pharmaceuticals).

## Figures and Tables

**Table 1:** Total yield and component composition of cold maceration of *Labisia pumila* leaf extract.

P (bar)	Solvent/Co-Solvent Type	Snyder’s Solvent Polarity Index ^a^	Total Yield (Y_TOT_) (% *g/g*) ± S.D.	Component Content (% *g/g* Extract) ± S.D.		
				Gallic Acid (GA)	Methyl Gallate (MG)	Caffeic Acid (CA)
1	*n*-Hexane	0.1	$1.18\pm 0.24{\;}^{\;f}\;$	*ND*	*ND*	*ND*
1	Ethanol	5.2	$3.10\pm 0.14^{\;e}$	0.09 ± 0.02	0.02 ± 0.01	0.02 ± 0.01
1	70% ethanol	6.3	$5.91\;\pm 0.25{\;}^{c}$	0.16 ±0.08	0.03 ± 0.01	0.81 ± 0.03
1	Methanol	6.6	$3.14\;\pm 0.37{\;}^{e}$	0.06 ± 0.02	0.01 ± 0.04	0.02 ± 0.01
1	50% ethanol	7.1	$6.95\;\pm 0.18{\;}^{b}$	0.13 ± 0.05	0.04 ± 0.03	0.64 ±0.07
1	70% methanol	7.3	$5.04\;\pm 0.21{\;}^{d}$	0.14 ± 0.03	0.02 ± 0.01	0.72 ± 0.03
1	50% methanol	7.8	$6.70\;\pm 0.20{\;}^{\mathrm{cd}}$	0.11 ± 0.01	0.02 ± 0.02	0.70 ±0.05
1	Water	9.0	$3.05\;\pm 0.08{\;}^{e}$	0.17 ± 0.07	0.01 ± 0.01	0.01 ± 0.01

(^a^) Snyder’s solvent polarity index [19]. The aqueous solvent mixture indexes were calculated from equation (*I*_A_/100 × *P*_A_) + (*I*_B_/100 × *P*_B_) where *I*_A_ and *I*_B_ are polarity index of solvents A and B, respectively, and *P*_A_ and *P*_B_ are the percentage of solvents A and B, respectively, in the solvent mixture. *ND*—Not Detected.; Values were expressed as mean ± SD. Different letters in the same column represent a significant difference (*p* < 0.05) within one category of Solvent/Co-solvent Type. (^b^) The highest yield and significantly different from other yields (^c^,^cd^,^d^,^e^,^f^).

**Table 2:** Total yield and component composition of Supercritical Fluid Extraction *Labisia pumila* leaf extract.

T (°C)	P (bar)	Solvent/Co-Solvent Type	Snyder’s Solvent Polarity Index ^a^	Total Yield (Y_TOT_) (% *g/g*) ± S.D.	Component Content (% *g/g* Extract) ± S.D.		
					Gallic Acid (GA)	Methyl Gallate (MG)	Caffeic Acid (CA)
60	200	-	-	$2.95\pm 0.80{\;}^{e}$	*ND*	*ND*	*ND*
60	200	Ethanol	5.2	$6.91\pm 0.18{\;}^{d}$	0.15 ± 0.09	0.13 ± 0.01	0.49 ± 0.05
60	200	70% ethanol	6.3	$13.90\pm 0.50{\;}^{b}$	0.30 ± 0.05	0.28 ± 0.03	1.11 ± 0.01
60	200	Methanol	6.6	$7.02\pm 0.75{\;}^{d}$	0.17 ± 0.03	0.10 ± 0.09	0.55 ± 0.01
60	200	50% ethanol	7.1	$14.71\pm 0.31{\;}^{b}$	0.19 ± 0.05	0.21 ± 0.01	1.00 ± 0.03
60	200	70% methanol	7.3	$11.01\pm 0.46{\;}^{c}$	0.26 ± 0.01	0.26 ± 0.02	0.99 ± 0.04
60	200	50% methanol	7.8	$14.00\pm 0.17^{\;b}$	0.14 ± 0.02	0.19 ± 0.06	1.03 ± 0.01
60	200	Water	9.0	$4.06\pm 0.10^{\;e}$	0.20 ± 0.07	0.09 ± 0.03	0.11 ± 0.01

(^a^) Snyder’s solvent polarity index [19]. The aqueous solvent mixture indexes were calculated from equation (*I*_A_/100 × *P*_A_) + (*I*_B_/100 × *P*_B_) where *I*_A_ and *I*_B_ are polarity index of solvents A and B, respectively, and *P*_A_ and *P*_B_ are the percentage of solvents A and B, respectively, in the solvent mixture. *ND*—Not Detected.; Values were expressed as mean ± SD. Different letters in the same column represent a significant difference (*p* < 0.05) within one category of Solvent/Co-solvent Type. (^b^) Highest value and significantly different from other (^c^,^d^,^e^).

**Table 3:** Antioxidant capability of *L. pumila* extracts and the reference standards (positive control).

Operating Condition	Code	Solvent/Co-Solvent Type	TPC (mgGAE/100 g)	FRAP (μmol Fe (II)/g)	IC_50_ (µg/mL)
Control antioxidant compounds	Ascorbic acid	-	$663.02\;\pm 2.90{\;}^{a}$	$650.39\;\pm 2.60{\;}^{a}$	$41.83\;\pm 0.28{\;}^{b}$
	BHT	-	$640.38\pm 1.40{\;}^{d}$	$628.18\pm 1.90{\;}^{d}$	$53.37\pm 0.39{\;}^{a}$
Reference antioxidant compounds	Gallic acid	-	$657.38\;\pm 3.10^{\;\mathrm{ab}}$	$644.85\;\pm 4.00^{\;\mathrm{ab}}$	$13.32\;\pm 0.37{\;}^{e}$
	Methyl gallate	-	$650.59\;\pm 2.80{\;}^{\mathrm{bc}}$	$638.20\;\pm 2.50^{\;\mathrm{bc}}$	$16.20\;\pm 0.46^{\;d}$
	Caffeic acid	-	$646.45\;\pm 4.20^{\;\mathrm{cd}}$	$634.14\pm 3.10{\;}^{\mathrm{cd}}$	$19.83\;\pm 0.57^{\;c}$
Maceration		*n*-Hexane	$197.17\;\pm 1.30^{\;h}$	$193.42\;\pm 2.80^{\;h}$	$367.83\;\pm 1.19^{\;a}$
		Ethanol	$415.21\pm 2.50{\;}^{f}$	$407.30\pm 2.40{\;}^{f}$	$113.68\pm 0.53^{\;c}$
		70% ethanol	$554.25\;\pm 2.60{\;}^{a}$	$543.69\;\pm 0.09{\;}^{a}$	$63.65\;\pm 0.32{\;}^{g}$
		Methanol	$330.51\;\pm 1.70{\;}^{g}$	$324.21\;\pm 3.50{\;}^{g}$	$137.05\;\pm 2.02{\;}^{b}$
		50% ethanol	$483.41\;\pm 0.90^{\;d}$	$474.20\;\pm 2.20{\;}^{d}$	$91.54\;\pm 0.25^{\;d}$
		70% methanol	$522.62\;\pm 1.90^{\;b}$	$512.664\;\pm 1.70{\;}^{b}$	$69.19\;\pm 0.31^{\;f}$
		50% methanol	$467.70\;\pm 3.10{\;}^{e}$	$458.79\;\pm 3.10{\;}^{e}$	$88.71\;\pm 0.52{\;}^{e}$
		Water	$500.48\;\pm 2.60{\;}^{c}$	$490.94\;\pm 4.10{\;}^{c}$	$88.17\;\pm 0.06{\;}^{e}$
SC–CO_2_		-	$266.12\;\pm 2.10{\;}^{e}$	$251.37\;\pm 6.20{\;}^{f}$	$245.58\;\pm 5.09^{\;a}$
		50% ethanol	$653.71\;\pm 2.70^{\;\mathrm{ab}}$	$617.48\;\pm 4.10{\;}^{\mathrm{ab}}$	$61.53\;\pm 0.01{\;}^{d}$
		50% methanol	$647.06\;\pm 1.60{\;}^{\mathrm{bc}}$	$611.20\;\pm 0.90{\;}^{\mathrm{bc}}$	$63.69\;\pm 0.21{\;}^{d}$
		70% ethanol	$659.35\;\pm 3.10{\;}^{a}$	$622.81\;\pm 3.10{\;}^{a}$	$45.61\;\pm 0.53{\;}^{e}$
		70% methanol	$651.04\;\pm 3.50^{\;b}$	$614.96\;\pm 5.30{\;}^{\mathrm{abc}}$	$47.90\;\pm 0.57{\;}^{e}$
		Ethanol	$622.41\pm 1.90^{\;c}$	$587.91\pm 1.70{\;}^{d}$	$73.34\;\pm 0.21{\;}^{c}$
		Methanol	$641.57\;\pm 4.20{\;}^{b}$	$606.01\;\pm 1.20{\;}^{c}$	$70.47\;\pm 0.52{\;}^{c}$
		Water	$567.03\;\pm 2.00^{\;d}$	$535.60\;\pm 1.90{\;}^{e}$	$79.35\;\pm 2.31{\;}^{b}$

Values were expressed as mean ± SD. Different letters in the same column represent a significant difference (*p* < 0.05) within one category of Solvent/Co-solvent Type. (^a^) Snyder’s solvent polarity index; (^b^) Highest yield and significantly different from other (^c^,^cd^,^d^,^e^,^f^).
